# Supplementary material for: A new frog of the Leptodactylus fuscus species group (Anura: Leptodactylidae), endemic from the South American Gran Chaco
Source: PeerJ. 2019 Oct 11;7:e7869. doi: 10.7717/peerj.7869 (PMC6791353; doi:10.7717/peerj.7869)
Supplement: Supplemental Information 1 — Species, sample locality (municipality or conservation unit, state/province and country, respectively), GenBank accession number (12S and 16S rDNA sequences) and source. [file peerj-07-7869-s001.docx]

| *Species* | Locality | 12S | 16S | Source |
| --- | --- | --- | --- | --- |
| *Leptodactylus apepyta* **sp. nov.** | El Ceibal, Tucumán, Argentina | MN153829 | MN153968 | This study |
|  | Estancia Aguada Siete, Boquerón, Paraguay | MN153830 | MN153969 | This study |
|  | El Cadillal, Tucumán, Argentina | MN153831 | MN153970 | This study |
|  | Las Lomitas, Formosa, Argentina | MN153832 | MN153971 | This study |
|  | Los Colorados, Salta, Argentina | MN153833 | MN153972 | This study |
|  | Laprida, Santiago del Estero, Argentina | MN153834 | MN153973 | This study |
|  | San Sebastián, Santa Cruz de La Sierra, Bolivia |  | JF789906 | Jansen et al. (2011) |
| *L. mystacinus* | APM Manso, Mato Grosso, Brazil | MN153836 | MN153975 | This study |
|  | Morro do Diabo, Sao Paulo, Brazil | MN153837 | MN153976 | This study |
|  | Jacarezinho, Paraná, Brazil | MN153838 | MN153977 | This study |
|  | Cristina, Minas Gerais, Brazil | MN153844 | MN153983 | This study |
|  | Petrolina, Goiás, Brazil | MN153848 | MN153987 | This study |
|  | Los Gigantes, Córdoba, Argentina | MN153835 | MN153974 | This study |
|  | PN El Palmar, Entre Ríos, Argentina | MN153839 | MN153978 | This study |
|  | Villa Bonita, Misiones, Argentina | MN153840 | MN153979 | This study |
|  | Paraje Caabí Poí, Corrientes, Argentina | MN153841 | MN153980 | This study |
|  | Jesús María, Córdoba, Argentina | MN153842 | MN153981 | This study |
|  | Paraná, Entre Ríos, Argentina | MN153843 | MN153982 | This study |
|  | San Vicente, Misiones, Argentina | MN153845 | MN153984 | This study |
|  | Felipe Yofré, Corrientes, Argentina | MN153849 | MN153988 | This study |
|  | Bañado de los Oliveras, Treinta y Tres, Uruguay | MN153846 | MN153985 | This study |
|  | Bella Unión, Artigas, Uruguay | MN153847 | MN153986 | This study |
| *L. albilabris* |  | KM091462 | KM091577 | de Sá et al. (2014) |
| *L. bufonius* |  | MN153827 | MN153966 | This study |
| *L. camaquara* |  | KM091462 | KM091578 | de Sá et al. (2014) |
| *L. cunicularius* |  | KY992467 | | de Carvalho et al. (Unpublished) |
| *L. cupreus* |  | MN153828 | MN153967 | This study |
| *L. didymus* |  | AY948953 | AY948957 | de Sá et al. (2005) |
| *L. elenae* |  | KM091466 | KM091582 | de Sá et al. (2014) |
| *L. fragilis* |  | KM091469 |  | de Sá et al. (2014) |
| *L. furnarius* |  | KY992486 | | de Carvalho et al. (Unpublished) |
| *L. fuscus* 1 |  |  | AY911275 | Camargo et al. (2006) |
| *L. fuscus* 2 |  | AY905712 | AY911281 | Camargo et al. (2006) |
| *L. fuscus* 4 |  | DQ283404 | | Frost et al. (2006) |
| *L. fuscus* 5 |  |  | AY911284 | Camargo et al. (2006) |
| *L. fuscus* 6 |  | AY905702 | AY911271 | Camargo et al. (2006) |
| *L. fuscus* 7 |  | AY905705 | AY911274 | Camargo et al. (2006) |
| *L. gracilis* |  | KM091471 | KM091587 | de Sá et al. (2014) |
| *L. jolyi* |  | KM091475 | KM091591 | de Sá et al. (2014) |
| *L. labrosus* |  | KM091477 | KM091593 | de Sá et al. (2014) |
| *L. laticeps* |  | KM091479 | KM091594 | de Sá et al. (2014) |
| *L. latinasus* |  | KM091480 | KM091595 | de Sá et al. (2014) |
| *L. longirostris* |  | KM091483 | KM091596 | de Sá et al. (2014) |
| *L. marambaiae* |  | KM091486 | KM091600 | de Sá et al. (2014) |
| *L. mystaceus* 1 |  | AY905717 | AY911286 | Camargo et al. (2006) |
| *L.* cf. *mystaceus* (*L. mystaceus* 3) |  | AY948954 | AY948958 | de Sá et al. (2005) |
| *L. notoaktites* |  | KM091504 | KM091604 | de Sá et al. (2014) |
| *L. plaumanni* |  | KM091493 | KM091609 | de Sá et al. (2014) |
| *L. poecilochilus* |  | KM091495 | KM091611 | de Sá et al. (2014) |
| *L. sertanejo* |  |  | KU495355 | Lyra et al. (Unpublished) |
| *L. syphax* |  | KM091501 | KM091618 | de Sá et al. (2014) |
| *L. tapiti* |  | KY992494 | | de Carvalho et al. (Unpublished) |
| *L. troglodytes* |  | KM091502 | KM091620 | de Sá et al. (2014) |
| *L. ventrimaculatus* |  | KM091503 | KM091621 | de Sá et al. (2014) |
| *L. myersi* |  | KM091487 | KM091601 | de Sá et al. (2014) |
| *L. pentadactylus* |  | KM091491 | KM091607 | de Sá et al. (2014) |
| *L. rhodomystax* |  | AY947869 | AY947855 | Heyer et al. (2005) |
| *L. rugosus* |  | KM09199 | KM091615 | de Sá et al. (2014) |
| *L. bolivianus* |  | KM091461 | HQ232831 | de Sá et al. (2014); Heyer et al., (Unpublished) |
| *L. chaquensis* |  | EF613179 | EF632055 | Yanek et al. (2006) |
| *L. macrosternum* |  | KM091485 | KM091599 | de Sá et al. (2014) |
| *L. latrans* |  | KM091490 | KM09160 | de Sá et al. (2014) |
| *L. natalensis* |  | KM091488 | KM091602 | de Sá et al. (2014) |
| *L. podicipinus* |  | EF613172 | EF632048 | Yanek et al. (2006) |
| *Hydrolaetare caparu* |  | KM091473 | KM091589 | de Sá et al. (2014) |
| *Physalaemus cuvieri* |  | AY843729 | | Faivovich et al. (2005) |
| *Engistomophs petersi* |  | EF011554 | | Boul et al., (2007) |
| *Hyalinobatrachium Fleischmanni* |  | DQ283453 | | Frost et al. (2006) |
