# Supplementary material for: A new frog of the Leptodactylus fuscus species group (Anura: Leptodactylidae), endemic from the South American Gran Chaco
Source: PeerJ. 2019 Oct 11;7:e7869. doi: 10.7717/peerj.7869 (PMC6791353; doi:10.7717/peerj.7869)
Supplement: Supplemental Information 2 [file peerj-07-7869-s002.docx]

*Leptodactylus albilabris* (Günther, 1859) – PUERTO RICO: San juan: Río Piedras (MNRJ 80255).

*Leptodactylus apepyta* **sp. nov.** **–** ARGENTINA: Chaco: LGE 15236, 32 km SE Comandancia Frías (24°48’03”S, 62°04’52”W, 178 m asl), collected on 19 December 2008 by Baldo D. and E. Krauczuk; LGE 12095, rout from Comandancia Frías (24°48’24”S, 61°38’43.5”W, 161 m asl), collected on 15 November 2015 by Baldo D., Duport Bru A.S. and J. Grosso; LGE 15250–1, 15276, 3,5 km SE El Pintado (24°39’12”S, 61°28’18”W, 155 m asl), collected on 28 January 2009 by Baldo D. and E. Krauczuk; LGE 15232, 31 km NE Fuerte Esperanza (25°02’40”S, 61°33’08”W, 156 m asl), collected on 20 December 2008 by Baldo D. and E. Krauczuk; LGE 15275, 15280, 16944, 5 km NE Misión Nueva Pompeya (24°51’46”S, 61°26’58”W, 154 m asl), collected on 31 January 2009 by Baldo D., Krauczuk E. and J. Lima; LGE 12290, 12298, 27.8 km SE Misión Nueva Pompeya (25°04’07.08”S, 61°15’21.66”W, 142 m asl), collected on 19 November 2015 by Baldo D., Duport Bru A.S., Grosso J., Lescano J. and E. Gallion; LGE 15240, near to Paraje El Colorado along Ruta Provincial Nº 61 (24°48’19”S, 61°26’29”W, 155 m asl), collected on 30 January 2009 by Baldo D. and E. Krauczuk; LGE 15227, 15272, 15274, 15279, 4 km NE Wichí (24°40’19”S, 61°27’39”W, 153 m asl), collected on 28 January 2009 by Baldo D. and E. Krauczuk; Formosa: LGE 9399, 6 km E Colonia Villafañe (26°11’51”S, 59°01’06.6”W, 76 m asl), collected on 23 November 2014 by Baldo D. and D. Cardozo; MACN 45794–6, Ingeniero Juarez, collected by Blotto B. and A. Sehinkman; LGE 8181, Laguna Yema (24°13’10.8”S, 61°12’40.4”W, 162 m asl), collected on 30 January 2014 by Baldo D., Boeris J.M., Brusquetti F. and J. Grosso; MACN 46207, 49856–64, collected on 11 January 1998 by Fernicola J.C., Faivovich J. and R. Lajmanovich; Jujuy: CENAI 1890, Yuto, by A. Barrio; LGE 11231–41, Zapla Forestal (24°14’40.05”S, 65°08’46.03”W), collected on January 2015 by M.S. Akmentins; Salta: LGE 15238, 3 km N Embarcación along Ruta Nacional Nº34 (23°10’53”S, 64°04’34”W), collected on 20 November 2007 by Baldo D. and R. de Sá; CENAI 9878, Hickmann, collected on March 1961 by S. Pierotti; MACN 36093, Laguna de Las Catas, Piquirenda, collected on October 1995 by Bosso A., Carrizo G., Giraudo A. and M. Viñas; LGE 15214, Los Colorados (24°35’00”S, 63°11’00”W), collected on 2 January 2002 by Barrionuevo S. and J.C. Moreta; Santiago del Estero: LGE 8384, La Loma along Ruta Provincial N°25 (27°43’03”S, 63°33’10.4”W, 143 m asl), collected on 4 February 2014 by Baldo D., Boeris J.M., Brusquetti F. and J. Grosso; LGE 15241, 15268, Laprida (28°22’56”S, 64°31’49”W, 207 m asl), collected on 28 December 2008 by Baldo D., Boeris J.M. and S. Rosset; CENAI 2125–6, Loreto, collected on 21 November 21 1966 by A. Barrio; MACN 27034, Nueva Esperanza, collected on 30 May 1968 by M. Rumboll; Tucumán: LGE 122, near to El Cadillal (26°39’24”S, 63°13’46”W), collected on 30 November 2009 by M.F. Vera Candioti; LGE15248–9, San Pablo, El Ceibal (26°52’00”S, 65°19’00”W), collected on 17 December 2002 by M.L. Ponssa; PARAGUAY: Boquerón: IIBP-H 1738, Estancia Aguada Siete, Ruta Joel Estigarribia (22°26’29.61”S, 61°45’42.58”W), collected on 26 October 2011 by Brusquetti F. and M.T.C. Thomé; IIBP-H 2308, 35.5 km NE Infante Rivarola (21°35’27.56”S, 62°07’06.42”W) collected on 5 January 2013 by Netto Sisa F. and F. Brusquetti; IIBP-H 2848–9, Puesto Militar Capitán Joel Estigarribia (22°36’30.02”S, 61°26’24.57”W), collected on 8 January 2014 by Netto Sisa F. and F. Brusquetti; Presidente Hayes: IIBP-H 728–9, 20 km W Concepción along Ruta Nacional N°5 (23°28’21.68”S, 57°38’07.8”W), collected on 12 October 2012 by Baldo D., Brusquetti F. de Sá R. and F. Netto Sisa.

*Leptodactylus bufonius* Boulenger, 1894 – ARGENTINA: Chaco: Wichí (LGE 12154–5); Corrientes: Paraje Perichón (LGE 13437–40); Formosa: Pirané (LGE 9390–2); Salta: Embarcación (LGE 4994–5); Santiago del Estero: Monte Quemado (LGE 13261–4); PARAGUAY: Presidente Hayes: Pozo Colorado (MNRJ 69308–11).

*Leptodactylus caatingae* Heyer and Juncá, 2003 – BRAZIL: Bahia: Carfanaum (CFBH 34577); Manoel Vitorino (CFBH 28147); Serra do Ramalho (CFBH 21003, 22072–4, 27674–9; 36162–3); Pernambuco: Serra Talhada (CFBH 31730).

*Leptodactylus camaquara* Sazima and Bokermann, 1978 – BRAZIL: Minas Gerais: Lima Duarte (MNRJ 76133), Serra do Cipó (LGE 2675, 2699); Santana do Riacho (MNRJ 38735–6); Santa Bárbara (MNRJ 72750–1).

*Leptodactylus cunicularius* Sazima and Bokermann, 1978 – BRAZIL: Minas Gerais: Sacramento (CFBH 36014); Santana do Riacho (CFBH 782, 30897, 39836–9, 39843).

*Leptodactylus cupreus* Caramaschi, Feio and São Pedro, 2008 – BRAZIL: Bahia: Porto Seguro (CFBH 32113–4); Espírito Santo: Cariacica (CFBH 23632–50); Linhares (CFBH 26359–60).

*Leptodactylus didymus* Heyer, García-Lopez and Cardoso, 1996 – BRAZIL: Mato Grosso: Paranaíta (CFBH 28616).

*Leptodactylus elenae* Heyer, 1978 – ARGENTINA: Chaco: General Güemes (LGE 11736, 11741, 12243); San Fernando (LGE 9798); Jujuy: Santa Bárbara (LGE 11742); Misiones: Candelaria (LGE 11737–8); San Ignacio (LGE 11814–5); Santa Fe: Vera (LGE 11739); BRAZIL: Mato Grosso: Guaporé (MZUSP 134043).

*Leptodactylus furnarius* Sazima and Bokermann, 1978 – ARGENTINA: Misiones: Candelaria (LGE 12854–8); San Javier (LGE 7889).

*Leptodactylus fuscus* (Schneider, 1799) – ARGENTINA: Chaco: Wichí (LGE 15376); corrientes: Ituzaingó (LGE 15373, 15378); Formosa: Pirané (LGE 15369); misiones: Candelaria (LGE 15380–1); Villa Bonita (LGE 16609–11); salta: Iruya (LGE 15304–5); BRAZIL: Pará: Santa Bárbara (LGE 2702).

*Leptodactylus gracilis* (Duméril and Bibron, 1840) – ARGENTINA: Córdoba: Alpa Corral (LGE 12309); Punilla (LGE 7771); Corrientes: Curuzú Cuatiá (LGE 10397); Ituzaingó (LGE 2239, 5191, 5203); Paraje Caabí Poí (LGE 9352); Misiones: Azara (LGE 9679); Profundidad (LGE 7273); Tucumán: Sierras de Medina (LGE 10238).

*Leptodactylus jolyi* Sazima and Bokermann, 1978 – BRAZIL: Minas Gerais: Santana do Riacho (CFBH 39817, 39819); São Roque de Minas (CFBH 36478–9, 40105–8); São Paulo: Cubatão (CFBH 11546).

*Leptodactylus laticeps* Boulenger, 1918 – ARGENTINA: Chaco: El Sauzal (LGE 15282); Madrejones (LGE 15293); Misión Nueva Pompeya (LGE 12083, 12111, 15289–90); Pozo del Anta (LGE 15292); Formosa: Ingeniero Juárez (LGE 15284–5); Salta: Coronel Juan Solá (LGE 14953).

*Leptodactylus latinasus* Jiménez de la Espada, 1875 – ARGENTINA: Chaco: Taco Pozo (LGE 11243, 11246–7, 11250); Wichí (LGE 12120, 12125); Corrientes: Curuzú Cuatiá (LGE 12597); Paraje Perichón (LGE11285, 11289); Salta: Manuel Elordi (LGE 10529).

*Leptodactylus longirostris* Boulenger, 1882 – BRAZIL: Amazonas: Manaus (CFBH 4224); Pará: Oriximiná (MNRJ 48137, 48139, 48142, 56678).

*Leptodactylus marambaiae* Izecksohn, 1976 – BRAZIL: Rio de Janeiro: Ilha da Marambaia (MNRJ 19950, 20088, 30932, 40743–6).

*Leptodactylus mystaceus* (Spix, 1824) – BRAZIL: Goiás: Minaçu (LGE 10607, 10634); São Paulo: Botucatú (CFBH 36374).

*Leptodactylus mystacinus* (Burmeister, 1861) – ARGENTINA: Buenos Aires: Felipe Solá (MZUSP 83282); Trenque Lauquen (MACN 25250); Chaco: Santa Sylvina (MACN 24020); Córdoba: Cerro Colorado (CENAI 4131–2); Cruz Chica (MACN 17790); Jesús María (LGE 15215, 15233); Los Gigantes (LGE 7773–4); Parque Siquiman (CENAI 3851, 3588, 4397–8); Pozo Verde (MACN 33440); Río Tercero (LGE 418–9, 15234); San Javier (MACN 7713); Santa Rosa de Calamuchita (CENAI 80-1,80-2, 80-3); Tanti (LGE 14999); Corrientes: Felipe Yofré (CENAI 2393–4); Garruchos (LGE 9072–4); Mburucuyá (MACN 13106, 13111); Paraje Caabí Poí (LGE 9343, 09356–9, 09386); San Carlos (MACN 29585, 29591); San Luís del Palmar (LGE 1620); Entre Ríos: Arroyo Urquiza (CENAI 3128); Chajarí (LGE 15201–8); Concepción del Uruguay (MACN 20054–5, 25175–6); Federal (LGE 18886–90); La Paz (MACN 46520, 47303–7); Paraná (LGE 15237); Parque Nacional El Palmar (LGE 1542–3); Pronunciamiento (MACN 27589–90); Villaguay (MACN 38112–4, 46313; LGE 18772–3); Vuelta de Obligado (MACN 30374); la pampa: Cerro Quemado (MACN 23269–72); Conhelo (MACN 6913); General Pico (MACN 18316, 19273, 19440–1, 20995); Realicó (CENAI 1319); Misiones: Andresito (LGE 7607–8, 7615–6, 7929, 14993–8, 15222–4, 15228, 15231, 15235, 15269); Aristóbulo del Valle (LGE 15212–3, 16461–3, 16483); Bernardo de Irigoyen (CENAI 6708–9, 6711–3, 6715, 6717, 7604–5); Camping Malvinas (LGE 5933, 6136–9); Colonia Victoria (LGE 15209–11); Dos de Mayo (CENAI 6592, 6594, 6597–601); Dos Hermanas (LGE 3952, 4508, 4511, 4959–60); El Soberbio (LGE 15273); Eldorado (CENAI 6498); Foerster (LGE 2284); Garupá (LGE 3873, 3961); General Belgrano (CENAI 7075); Itacaruaré (LGE 7890); La Corita (LGE 19987–91); Ñu Pyahú (LGE 15216–8, 15281); Paraje Cabureí (LGE 15271); Paraje Macaca (LGE 7606); Parque Nacional Iguazú (LGE 3982, 3995, 3997, 6852, 6873, 6936, 6955, 6965); Parque Provincial Moconá (LGE 15219–21); Parque Provincial Piñalito (LGE 264–5, 1695–6, 7613, 20431); Parque Provincial Urugua-í (LGE 06923, 15239, 15270); Posadas (LGE 14–5); Puerto Esperanza (LGE 4677–9, 6145–50, 6153, 6157–8, 6162–3, 6169–73); Puerto Iguazú (LGE 15225–6); Puerto Libertad (LGE 6913–4, 6940, 6944, 6946); Puerto Londero (LGE 19952–6); Puerto Panambí (LGE 7903–4); Reserva de Biosfera Yabotí (LGE 12410–1); Reserva Natural Estricta San Antonio (LGE 3996, 3998); Reserva San Jorge (LGE 6954); San Ignacio (LGE 1565); San Sebastián de la Selva (LGE 15242–6, 15277–8); San Vicente (LGE 15247); Santiago de Liniers (LGE 7063, 7075–6); Villa Bonita (LGE 4237–9, 4258–61, 16560–1, 16568); San Luís: Candelaria (LGE 20859–64, 20980–7); Junín (MACN 40507, 45113, 46206–7, 49168–9); Justo Daract (CENAI S/N); Quines (LGE 20954–7); Santa Rosa de Conlara (MACN 36675); Villa Larca (MACN 46254); Santa Fe: Las Colonias (MACN 36810); Las Rosas (MACN 23704–9), 24226–32,); Los Nogales (MACN 24564–73); Recreo (MACN 17363). BRAZIL: Bahía: Caetité (CFBH 21084); Gruta do Caiçara (MTR 24976); Guaratinga (MNRJ 25675); Iramaia (MNRJ 87639–40); Itamaraju (MNRJ 46851); Jequié (CFBH 32248); Maracás (MZUSP 83593–5); Miguel Calmon (CFBH 34631–3); Monte Alegre (MTR 38717); Morro do Chapéu (MNRJ 91556); Mucuri (CFBH 5540); Palmeiras (CFBH 27906, 30126–8, 30187, 30189); Distrito Federal: Brazilia (MCP 12777; MNRJ 12423, 2714); Espírito Santo: Cachoeira do Itapemirim (MZUSP 142811); Divinolândia (CFBH 4899); Linhares (MNRJ 35004; CFBH 19438); Mimoso do Sul (MNRJ 66170–2); Goiás: Colinas do Sul (MNRJ 86010); Luzianía (MZUSP 140857, 140863; MTR 11494–5); Minaçu (LGE 10541–2; MCP 2345–7, 2369, 2606, 2932–3, 2935, 2937–45; MNRJ 20167–73, 20176–86); Palmeiras de Goiás (CFBH 26089, 26118); Petrolina (MTR 8486); Pirenópolis (MNRJ 85549–50); UHE Corumbá (MZUSP 140858–60,140864–7, 140948, 140951, 140955–6, 140958, 140961, 140965, 140969–70, 140972, 140976–8, 140980, 140982–3); UHE Serra da Mesa (MTR 6845; MZUSP 89747-89807, 89754, 89756, 89763, 89772, 89776, 89782–4, 89787–9, 89793, 89798, 89807); Mato Grosso: Alto Taquari (PHV 2568); Barra do Garças (PHV 2304); Lambari D'Oeste (MNRJ 87245); Rosario D'oeste (MNRJ 87158–9; MZUSP 149016–7); Tangará da Serra (MCP 05474); Tesouro (MZUSP 141806–9); UHE Manso (MZUSP 100956–66, 100968–71, 100973–7); Vila Bela da Santíssima Trindade (MZUSP 148939–41; MNRJ 90020); Mato Grosso do Sul: Aquidauana (MZUSP 15300); Três Lagoas (CFBH 13596, 19610, 24037–8, 24043, 28573); Minas Gerais: Aiuruoca (MZUSP 142474); Araxá (CFBH 11570–1); Buritís (MZUSP 25069); Catas Altas (MNRJ 60475, 72831); Cristália (MNRJ 40584); Cristina (CFBH 33553–4); Furnas (CFBH 17348–9); Grão Mogol (CFBH 10221–2; MNRJ 88950, 88956); João Pinheiro (MNRJ 38815, 45223, 45225); Juiz de Fora (MNRJ 90269–70); Lagoa Santa (MZUSP 015877); Lagoa Santa (MNRJ 1050); Lima Duarte (MNRJ 33043); Marliéria (MTR 17596); Ponta Nova (CFBH 42708) (CFBH 42734); Santana do Riacho (CFBH 39840; MNRJ 38738); Santana do Rio Preto (MTR 23676, 23786, 23805); São Gonçalo do Rio Abaixo (MZUSP 143535); Serra do Cipó (MTR 20315, 20321, 20354, 20393); Turmalina (MNRJ 34493–6); Volta Grande (MNRJ 43647); paraná: Boa Vista da Aparecida (MCP 03606); Maringá (CFBH 17243–4); Ortigueira (MCP 12824); Pinhão (MCP 2049–51); Santo Antônio da Platina (MZUSP 024155); Rio de Janeiro: Arraial do Cabo (MNRJ 45739); Cabo Frío (MNRJ 47537); Cachoeiras de Macacu (MNRJ 86720–1); Carapebus (MNRJ 66430, 68450, 70495, 87981–9, 89254–5, 90173–4); São Conrado (MZUSP 60852); Iguaba Grande (MNRJ 56606–8, 59404); Itaperuna (MNRJ 84229–30); Macaé (CFBH 42142; MNRJ 75169, 90172); Maricá (MNRJ 77818); Niterói (MNRJ 51923); Rio Claro (MNRJ 91466); São João da Barra (MNRJ 54947–55); Sao Joao Marcos (MNRJ 91467); Saquarema (MNRJ 25448–9); Silva Jardim (MNRJ 73427–8); Teresópolis (CFBH 42770; MNRJ 5411); Rio Grande do Sul: Alegrete (UFRGS 2722, 4789); Alpestre (UFRGS 6011); Amaral Ferrador (UFRGS 2663); Arambare (MCP 04212); Bom Progresso (MCP 12305); Caçapava do Sul (MCP 3231, 10799; ZUFSM 5169, 5184); Cachoeira do Sul (UFRGS 755); Campo Novo (ZUFSM 4396, 04401); Candiota (LGE 10543; MCP 4243–4, 4299, 4406, 12620, 12621); Canela (CFBH 9535, 9536); Cerro Largo (UFRGS 3390); Cotiporã (UFRGS 4594); Cristal (MCP 10855); Derrubadas (MCP 1583, 2574, 4372; UFRGS 2202, 4339–40; ZUFSM 4473, 4525–6, 4551, 4592); Dois Irmãos das Missões (MCP 9775–6); Dom Feliciano (ZUFSM 3008–13); Dom Pedrito (MCP 10004, 10892, 10894, 10896–8, LGE 10544); Eldorado do Sul (UFRGS 1702); Eugenio de Castro (UFRGS 4812); Garruchos (MCP 793); Gravataí (MCP 08341); Guaporé (UFRGS 5117); Ibarama (ZUFSM 4086, 4268); Itá (ZUFSM 2370–1, 2377); Jaguarão (ZUFSM 10614); Lavras do Sul (UFRGS 2640; ZUFSM 10562, 10564–5); Machadinho (CFBH 6954–5, 6959); Manoel Viana (UFRGS 4247, 4450; ZUFSM 2432); Nonoai (UFRGS 1394); Pelotas (UFRGS 3885); Pinhal Grande (ZUFSM 4066); Salvador do Sul (CFBH 38243; UFRGS 6108); Santa Cruz do Sul (MCP 7256); Santa María (MNRJ 18762; ZUFSM 13, 57, 60–2, 69–72, 75, 87, 99–100, 106, 110, 121, 136, 138, 167, 320, 335, 356, 373, 384, 425, 431, 452, 611, 651, 668, 674, 707, 887, 907, 1622, 1668, 1715, 1737, 1983, 3126, 4157); Santana do Livramento (UFRGS 2856, 2871, 2878, 3737–40, 3776–7, 3830, 6220–3; ZUFSM 8460–2, 8464); São Francisco de Assis (ZUFSM 4289); São Gabriel (ZUFSM 4730, 4740, 9168, 9400, 9404, 9432, 9437, 9445, 9495, 9500, 9509, 9510, 9513, 9673–4, 9686, 9693, 9758, 9793, 9855, 10459, 11720); São Jeronimo (MCP 08208); São Lourenço do Sul (UFRGS 5341, 5369); São Luiz Gonzaga (ZUFSM 11781); São Vicente do Sul (ZUFSM 11406, 11426, 11528, 11531, 11545, 11548, 11556); Sentinela do Sul (MCP 10867); Tenente Portela (MCP 4543); Viamão (MCP 10689); Santa Catarina: Arvoredo (UFRGS 5177); Campos Novos (CFBH 13621); Itá (MCP 648–9, 8669; UFRGS 7168); Nova Teutônia (MZUSP 8694–8); São Domingos (CFBH 3845); Xaxim (MCP 9764); São Paulo: Angatuba (CFBH 23137); Assis (CFBH 18834, 18860, 20060–1, 38397); Barueri (MNRJ 74033); Bauru (CFBH 19764–8); Botucatu (CFBH 406, 36360, 36363; MNRJ 329–31, 398–9, 430, 434–6, 19309–13, 69321–3, 70632–5, 73703, 73722, 73871–81, 74513, 75224, 81621–2, 82058–9, 83437–40); Brotas (CFBH 9801–4, 9832–3); Buri (MZUSP 134834–7); Campinas (CFBH 999–1000, 34947, 35037, 35404–5, 38081); Corumbataí (CFBH 1339); Descalvado (CFBH 3994–5, 4002, 4008, 4538–9, 4726, 7603–9); Eugenio Lefevre (MZUSP 14907); Iperó (MTR 15558); Itapeva (MZUSP 138177); Itararé (MZUSP 138161, 138163, 138165, 138167–9, 138175–6); Itirapina (CFBH 6518, 9787–8, 9800, 9811, 9813–20); Itu (MZUSP 83285); Jaguaré (MZUSP 131608); Jaguariúna (MZUSP 143927); Lençóis Paulista (CFBH 39577, 39579); Limeira (MZUSP 143917); Luis Antônio (CFBH 33098–119, 38543); Mogi das Cruzes (CFBH 6585); Patrocínio Paulista (MNRJ 91251; CFBH 43296–7); Piquete (CFBH 35792); Piracicaba (CFBH 3829); Pontes Geral (CFBH 39886); Ribeirão Branco (CFBH 2499); Rio Claro (CFBH 6432–5, 7129–30, 7615); Santa Bárbara d'Oeste (MZUSP 154604–5); Santa Branca (MZUSP 25456); Santa Rita do Passa Quatro (CFBH 38859); Santana do Parnaíba (CFBH 9995); São Carlos (CFBH 26887, 26889–90); São Joaquim da Barra (CFBH 43387); Serra da Bocaina (MZUSP 53033, 53206–9, 53214–5); Tapiraí (CFBH 23332); Teodoro Sampaio (CFBH 10083–4, 10088, 10090, 18299, 18307, 18386); Tocantins: Peixe (MZUSP 129756–61); São Salvador (MZUSP 142134); PARAGUAY: Amambay: Estancia Pirá Potrero (IIBP-H 1085–6); Asunción: Asunción (MNRJ 400); Caaguazú: Caaguazú (MNRJ 70640–1); Canindeyú: Reserva Mbaracayú (IIBP-H 50, 72); Central: San Lorenzo (IIBP-H 254); Chaco: La Patria (IIBP-H 2308); Itapúa: Parque Nacional San Rafael (IIBP-H 1394); Reserva Guyrá-Retá (IIBP-H 1999); Yatai (IIBP-H 1185); Presidente Hayes: Concepción (IIBP-H 728–9); URUGUAY: Artigas: Bella Unión (LGE 15257); Canelones: Canelones (MZUSP 22640–1); Lavalleja: Minas (MZUSP 50220); Maldonado: Maldonado (MZUSP 83296); Montevideo: Cerro de Montevideo (CENAI 6300–1); Santiago Vázquez (LGE 15252–4); Río Negro: M´bopicúa (LGE 15259); Salsipuedes (CENAI 6302); Río Negro (LGE 15264); Rocha: Rocha (LGE 15262); La Pedrera (LGE 15263); Laguna de Rocha (LGE 15256); Punta del Diablo (LGE 15265); Salto: Termas del Arapey (LGE 15258); San José: Colonia Delta (LGE 15267); Treinta y Tres: Arroyo Tigre (LGE 19715); Bañado de los Oliveras (LGE 15260–1); Cementerio de los Fleitas (LGE 15266); Quebrada de los Cuervos (LGE 15255).

*Leptodactylus notoaktites* Heyer, 1978 – BRAZIL: Paraná: Matinhos (MNRJ 86417); Santa Catarina: São Bento de Sul (MNRJ 48296); São Paulo: Botucatu (MNRJ 83265), Jacupiranga (MNRJ 68322); Riberao Branco (MNRJ 17653, 19347–50); Tocantins: Pedro Alfonso (MNRJ 87663).

*Leptodactylus plaumanni* Ahl, 1936 – ARGENTINA: Misiones: Bernardo de Irigoyen (LGE 3375, 3386); Campo Arminda (LGE 5086, 5104); Colonia Taranco (LGE 3543, 3700); Parque Provincial Piñalito (LGE 9673–4); Puerto Londero (LGE 9675); San Pedro (LGE 9666).

*Leptodactylus sertanejo* Giaretta and Costa, 2007 – BRAZIL: Bahia: São Desidério (CFBH 20558).

*Leptodactylus spixi* Heyer, 1983 – BRAZIL: Bahia: Ilhéus (CFBH 27713, 27840, 39429); Uruçuca (CFBH 32530, 32441, 34055, 35738); Espírito Santo: Linhares (CFBH 5791, 25154, 26270, 26328, 26493); Mimoso do Sul (CFBH 25499); Muniz Freire (CFBH 4056, 4110); Rio de Janeiro: Rio de Janeiro (CFBH3 9683); Seropédica (CFBH 26352).

*Leptodactylus syphax* Bokermann, 1969 – BRAZIL: Goiás: Alto Paraíso (CFBH 6804–5, 25877); Minaçu (LGE 10626–7); Palmeiras de Goiás (CFBH 26085, 26122); Maranhão: Estreito (CFBH 18037); Mato Grosso do Sul: Bodoquena (CFBH 3840); Minas Gerais: Sacramento (CFBH 36539); Santana do Riacho (CFBH 798, 39835); Tocantins: Aurora de Tocantins (CFBH 39543, 35946).

*Leptodactylus tapiti* Sazima and Bokermann, 1978 – BRAZIL: Goiás: Alto do Paraíso de Goiás (CFBH 39586–90).

*Leptodactylus troglodytes* Lutz, 1926 – BRAZIL: Alagoas: Coruripe (MNRJ 38163); Ceará: Aracatí (MNRJ 88503); Bahia: Caetité (MNRJ 43915–6); Minas Gerais: Grao Mogol (MNRJ 88740); Pernambuco: Caruaru (MNRJ 38658); piauí: Riacho Frio (MNRJ 87635); Rio Grande do Norte: Mossoró (MNRJ 38581–3, 38599–600).
