# Supplementary material for: A new frog of the Leptodactylus fuscus species group (Anura: Leptodactylidae), endemic from the South American Gran Chaco
Source: PeerJ. 2019 Oct 11;7:e7869. doi: 10.7717/peerj.7869 (PMC6791353; doi:10.7717/peerj.7869)
Supplement: Supplemental Information 3 — Locality, voucher, and source. Uv, unvouchered specimen. [file peerj-07-7869-s003.docx]

| **Sound file** | **Locality** | **Voucher** | **Source** |
| --- | --- | --- | --- |
| *Leptodactylus apepyta* **sp. nov.** |  |  |  |
| LGE-B 001 | El Cadillal, Tucumán, Argentina. | Uv | Recorded in field |
| LGE-B 002 | El Ceibal, San Pablo, Tucumán, Argentina. | Uv | Recorder in field |
| LGE-B 003 | El Ceibal, San Pablo, Tucumán, Argentina, | Uv | Recorder in field |
| LGE-B 004 | Las Lomitas, Formosa, Argentina. | LGE 8085 | Recorded in field |
| LGE-B 005 | Presidente Hayes, Paraguay. | IIBP-H 728 | Recorded in field |
| *Leptodactylus mystacinus* |  |  |  |
| LGE-B 006 | Colonia Victoria, Misiones, Argentina. | LGE 15210 | Recorded in field |
| LGE-B 007 | Colonia Victoria, Misiones, Argentina. | LGE 15211 | Recorded in field |
| LGE-B 008 | Rosario, Santa Fe, Argentina. | Uv | Recorded in field |
| LGE-B 009 | Rosario, Santa Fe, Argentina. | Uv | Recorded in field |
| LGE-B 010 | Rosario, Santa Fe, Argentina. | Uv | Recorded in field |
| LGE-B 011 | Rosario, Santa Fe, Argentina. | Uv | Recorded in field |
| LGE-B 012 | Rosario, Santa Fe, Argentina. | Uv | Recorded in field |
| LGE-B 013 | Rosario, Santa Fe, Argentina., | Uv | Recorded in field |
| LGE-B 014 | Rosario, Santa Fe, Argentina. | Uv | Recorded in field |
| LGE-B 015 | Santiago Vazquez, Montevideo, Uruguay. | Uv | Recorded in field |
| LGE-B 016 | Ramallo, Buenos Aires, Argentina. | Uv | Recorded in field |
| LGE-B 017 | Ramallo, Buenos Aires, Argentina. | Uv | Recorded in field |
| LGE-B 018 | Ramallo, Buenos Aires, Argentina. | Uv | Recorded in field |
| STE-007 | Entre Ríos, Argentina | – | Ecoregistros |
| STE-013 | Entre Ríos, Argentina. | – | Ecoregistros |
| FNVJ_13087 | Pozo Verde, Córdoba, Argentina. | – | FNJV UNICAMP |
| FNVJ_31047 | Campinas, São Paulo, Brazil. | – | FNJV UNICAMP |
| FNVJ_31048 | Campinas, São Paulo, Brazil. | – | FNJV UNICAMP |
| FNVJ_31049 | Santiago Vazquez, Montevideo, Uruguay. | – | FNJV UNICAMP |
| FNVJ_31502 | Oberá, Misiones, Argentina. | – | FNJV UNICAMP |
| FNVJ_32074 | Campinas, São Paulo, Brazil. | – | FNJV UNICAMP |
| FNVJ_32150 | Valinhos, São Paulo, Brazil. | – | FNJV UNICAMP |
| FNVJ_32517 | Valinhos, São Paulo, Brazil. | – | FNJV UNICAMP |
| FNVJ_32518 | Valinhos, São Paulo, Brazil. | – | FNJV UNICAMP |
| FNVJ_33149 | Campinas, São Paulo, Brazil. | – | FNJV UNICAMP |
| FNVJ_34064 | Piracicaba, São Paulo, Brazil. | – | FNJV UNICAMP |
| FNVJ_36549 | Trancoso, Bahia, Brazil. | – | FNJV UNICAMP |
| FNVJ_36550 | Trancoso, Bahia, Brazil. | – | FNJV UNICAMP |
| 16682 | Derrubadas, Rio Grande do Sul, Brazil. | – | FonoZoo |
| 16709 | Derrubadas, Rio Grande do Sul, Brazil. | – | FonoZoo |
| 16980 | Candiota, Rio Grande do Sul, Brazil. | – | FonoZoo |
| 17019 | Derrubadas, Rio Grande do Sul, Brazil. | – | FonoZoo |
| 17276 | Santa Cruz do Sul, Rio Grande do Sul, Brazil. | – | FonoZoo |
| 17689 | Seará, Santa Catarina, Brazil. | – | FonoZoo |
| 21082 | Bella Vista, Corrientes, Argentina. | – | FonoZoo |
| 21083 | Bella Vista, Corrientes, Argentina. | – | FonoZoo |
| 21084 | Bella Vista, Corrientes, Argentina. | – | FonoZoo |
| 21085 | Bella Vista, Corrientes, Argentina. | – | FonoZoo |
| MNVOC031 | Catas Altas, Minas Gerais, Brazil. | – | MNRJ |
| MNVOC046-05 | Pirenópolis, Goiás, Brazil. | – | MNRJ |
| MNVOC046-07 | Pirenópolis, Goiás, Brazil. | – | MNRJ |
