# Supplementary figures and images for: A new frog of the Leptodactylus fuscus species group (Anura: Leptodactylidae), endemic from the South American Gran Chaco
Source: PeerJ. 2019 Oct 11;7:e7869. doi: 10.7717/peerj.7869 (PMC6791353; doi:10.7717/peerj.7869)

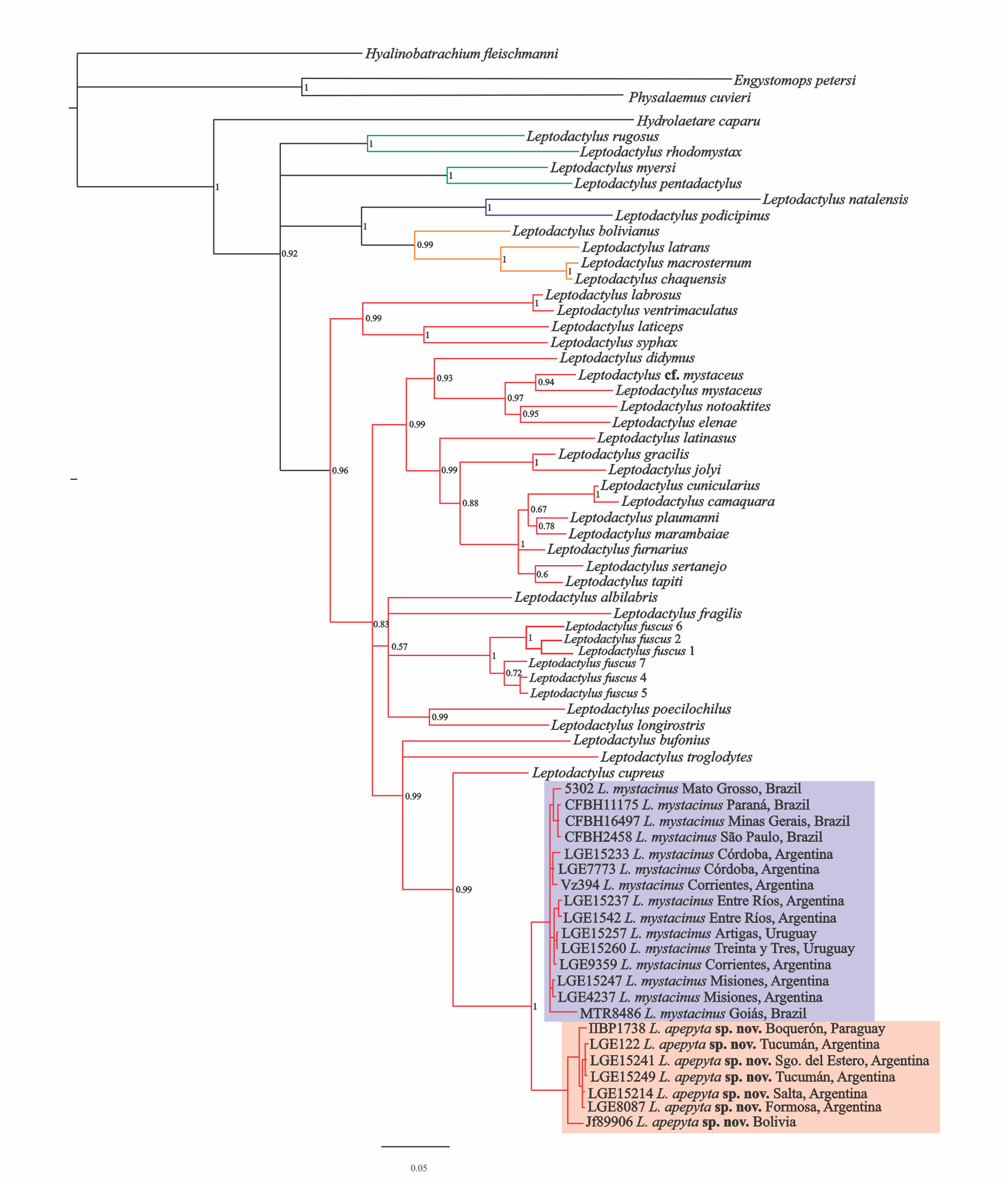

Supplement: Supplemental Information 4 — The 50% majority rule consensus tree from Bayesian inference analysis of concatenated mitochondrial fragments (16S and 12S rDNA). Posterior probabilities greater than 0.7 are shown on the nodes. Asterisks indicate posterior probability of 1.0. [file peerj-07-7869-s004.png]

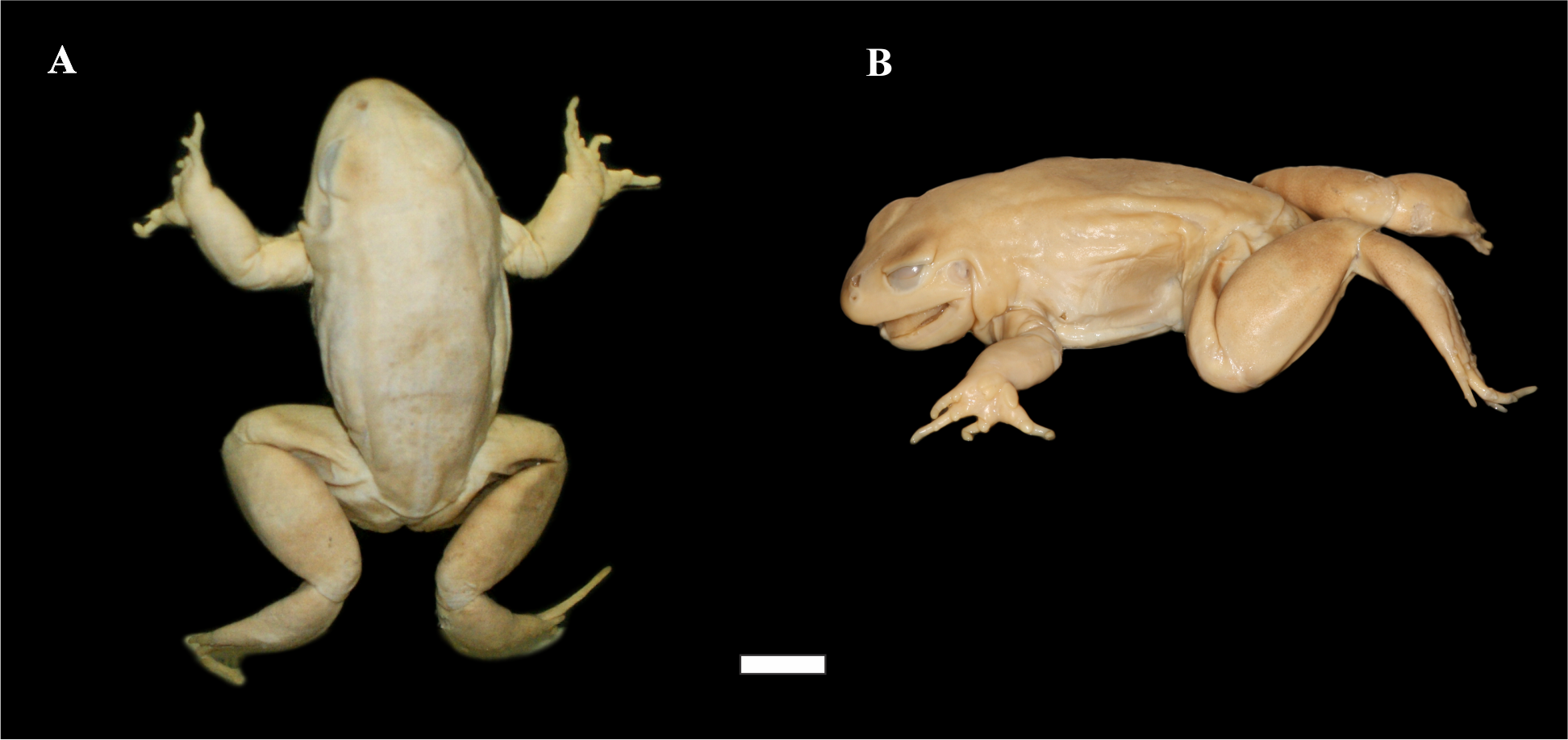

Supplement: Supplemental Information 6 — (A) Dorsal and (B) lateral views of the head of the holotype (MLU, unnumbered male). Scale bar = 10 mm. Photo: Axel Kwet. [file peerj-07-7869-s006.png]

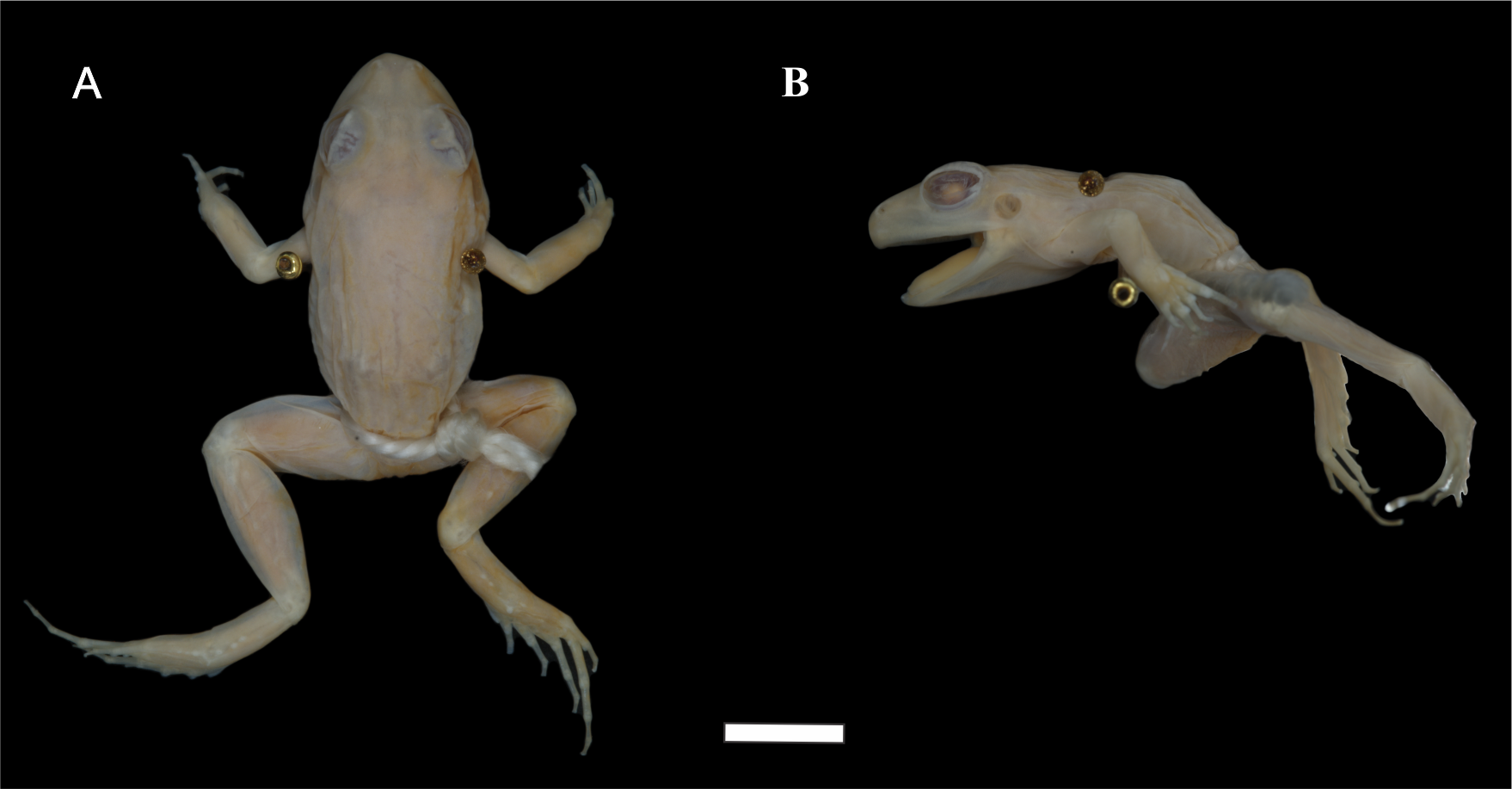

Supplement: Supplemental Information 7 — (A) Dorsal and (B) lateral views of the head of the lectotype (USNM 31302). Scale bar = 1 mm. SVL 17.26 mm. Photo: Esther M. Langan. [file peerj-07-7869-s007.png]

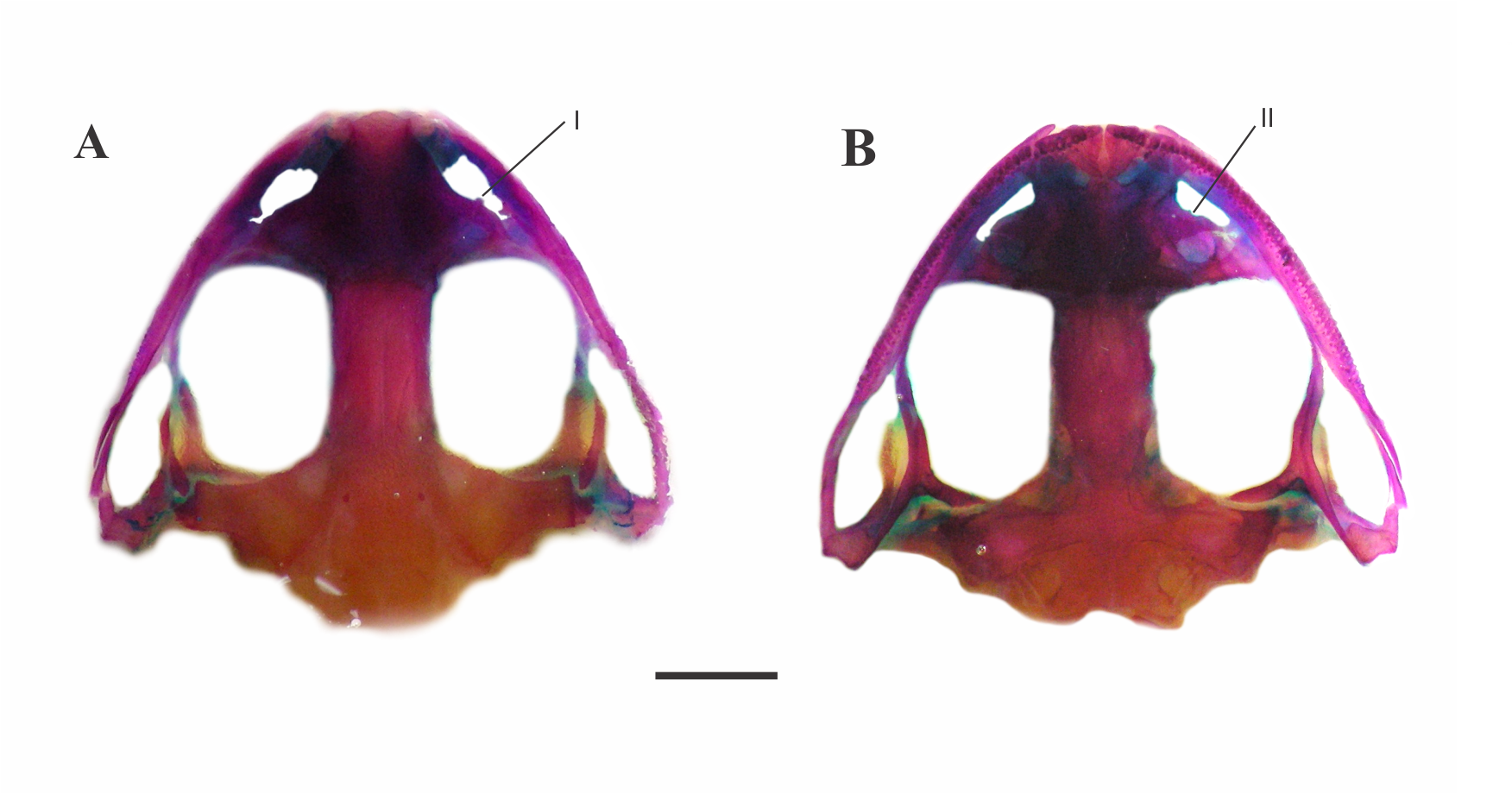

Supplement: Supplemental Information 9 — (A) Dorsal and (B) ventral view (LGE 15207). Bones showing differences from L. apepyta sp. nov. I. Nasal. II. Lateral edges of the middle ramus of prevomers. Scale bar = 2 mm. Photo: Rosio Schneider. [file peerj-07-7869-s009.png]
