# Supplementary material for: A new frog of the Leptodactylus fuscus species group (Anura: Leptodactylidae), endemic from the South American Gran Chaco
Source: PeerJ. 2019 Oct 11;7:e7869. doi: 10.7717/peerj.7869 (PMC6791353; doi:10.7717/peerj.7869)
Supplement: Supplemental Information 5 [file peerj-07-7869-s005.docx]

|  | Species | 1 | 2 | 3 | 4 | 5 | 6 | 7 | 8 | 9 | 10 | 11 | 12 | 13 | 14 | 15 | 16 | 17 | 18 | 19 | 20 | 21 | 22 | 23 | 24 | 25 | 26 | 27 | 28 | 29 |
| --- | --- | --- | --- | --- | --- | --- | --- | --- | --- | --- | --- | --- | --- | --- | --- | --- | --- | --- | --- | --- | --- | --- | --- | --- | --- | --- | --- | --- | --- | --- |
| 1 | *Leptodactylus apepyta* **sp. nov** | 0–1.3 |  |  |  |  |  |  |  |  |  |  |  |  |  |  |  |  |  |  |  |  |  |  |  |  |  |  |  |  |
| 2 | *L. mystacinus* | 3–4.4 | 0–2 |  |  |  |  |  |  |  |  |  |  |  |  |  |  |  |  |  |  |  |  |  |  |  |  |  |  |  |
| 3 | *L. cupreus* | 5.7–5.9 | 4.8–5.9 | – |  |  |  |  |  |  |  |  |  |  |  |  |  |  |  |  |  |  |  |  |  |  |  |  |  |  |
| 4 | *L. troglodytes* | 8.9–9.2 | 7.8–8.7 | 9.2 | – |  |  |  |  |  |  |  |  |  |  |  |  |  |  |  |  |  |  |  |  |  |  |  |  |  |
| 5 | *L. bufonius* | 7.4–7.6 | 7.2–8.5 | 8.1 | 11.1 | – |  |  |  |  |  |  |  |  |  |  |  |  |  |  |  |  |  |  |  |  |  |  |  |  |
| 6 | *L marambaiae* | 7.6–8.3 | 7.4–8.3 | 7.9 | 11.3 | 9.2 | – |  |  |  |  |  |  |  |  |  |  |  |  |  |  |  |  |  |  |  |  |  |  |  |
| 7 | *L. plaumanni* | 7.9–8.7 | 7.7–8.5 | 8.7 | 11.8 | 9.4 | 2.4 | – |  |  |  |  |  |  |  |  |  |  |  |  |  |  |  |  |  |  |  |  |  |  |
| 8 | *L. sertanejo* | 8.1–8.7 | 8.1–8.9 | 9.2 | 11.6 | 10.5 | 2.8 | 3.5 | – |  |  |  |  |  |  |  |  |  |  |  |  |  |  |  |  |  |  |  |  |  |
| 9 | *L. tapiti* | 8.1–8.7 | 8.5–9.4 | 8.9 | 10.7 | 10.3 | 3.1 | 3.7 | 2.6 | – |  |  |  |  |  |  |  |  |  |  |  |  |  |  |  |  |  |  |  |  |
| 10 | *L. furnarius* | 7.6–8.3 | 7.4–8.1 | 8.5 | 10.7 | 9.6 | 3.1 | 4.4 | 3.1 | 2.8 | – |  |  |  |  |  |  |  |  |  |  |  |  |  |  |  |  |  |  |  |
| 11 | *L. camaquara* | 12–12.5 | 11.2–11.8 | 12 | 15.1 | 13.1 | 6.8 | 7.9 | 8.5 | 7.4 | 7 | – |  |  |  |  |  |  |  |  |  |  |  |  |  |  |  |  |  |  |
| 12 | *L. cunicularius* | 9.6–10 | 8.9–9.6 | 10 | 12.9 | 10.9 | 4.4 | 5.5 | 6.3 | 5.2 | 5.2 | 3.1 | – |  |  |  |  |  |  |  |  |  |  |  |  |  |  |  |  |  |
| 13 | *L. gracilis* | 9.4–9.8 | 9.1–10 | 7.9 | 11.3 | 10.7 | 7.4 | 8.5 | 8.1 | 8.3 | 8.1 | 11.4 | 8.5 | – |  |  |  |  |  |  |  |  |  |  |  |  |  |  |  |  |
| 14 | *L. jolyi* | 10.5–10.9 | 11.1–12 | 9.4 | 12.5 | 11.6 | 9.7 | 9.9 | 10.1 | 10.3 | 9.8 | 13.2 | 10.7 | 3.5 | – |  |  |  |  |  |  |  |  |  |  |  |  |  |  |  |
| 15 | *L. elenae* | 10–10.4 | 9.4–10 | 9 | 13 | 10 | 7 | 7.9 | 8.1 | 8.3 | 8.5 | 11.6 | 9.2 | 8.1 | 10.3 | – |  |  |  |  |  |  |  |  |  |  |  |  |  |  |
| 16 | *L. notoaktites* | 9.4–10.1 | 8.8–9.9 | 8.6 | 11.2 | 9.6 | 7.9 | 8.8 | 8.3 | 9 | 8.8 | 11 | 9.4 | 10 | 11.4 | 8.5 | – |  |  |  |  |  |  |  |  |  |  |  |  |  |
| 17 | *L.* cf. *mystaceus* | 10.1–10.7 | 8.9–9.6 | 9.9 | 12.5 | 11.2 | 8.1 | 8.4 | 8.8 | 9.2 | 8.8 | 11.9 | 9.9 | 9.6 | 11.9 | 8.1 | 9.3 | – |  |  |  |  |  |  |  |  |  |  |  |  |
| 18 | *L. didymus* | 9.6–10.3 | 9.6–11 | 9.7 | 12.7 | 11 | 8.8 | 9.2 | 9 | 9.4 | 9.6 | 13 | 10.9 | 9.7 | 11.2 | 9 | 9.2 | 5.1 | – |  |  |  |  |  |  |  |  |  |  |  |
| 19 | *L. mystaceus* | 10.6–11 | 9.1–10.6 | 10.1 | 12.3 | 13.2 | 8.8 | 9.3 | 9.2 | 9.7 | 8.8 | 12.8 | 11 | 9 | 10.8 | 10.1 | 10 | 5.3 | 6.9 | – |  |  |  |  |  |  |  |  |  |  |
| 20 | *L. latinasus* | 9.4–9.8 | 9.2–10.5 | 10.1 | 11.2 | 10.3 | 9.2 | 9.2 | 10 | 9.6 | 9.4 | 11.8 | 10.3 | 9.2 | 10.3 | 9.8 | 9.4 | 10.3 | 10.5 | 11.2 | – |  |  |  |  |  |  |  |  |  |
| 21 | *L. fuscus* | 6.5–8.7 | 5.9–8.3 | 5.9–7.4 | 8.7–9.6 | 7.2–8.1 | 6.8–7.9 | 7.6–8.5 | 6.8–8.1 | 7.2–8.1 | 6.8–8.1 | 11.2–12.3 | 8.7–9.6 | 7.4–8.9 | 9–10.7 | 7–8.1 | 2.4–4.8 | 8.1-9.7 | 8.6–10.1 | 9.3–9.7 | 7.9–9.2 | 0.4–3.5 |  |  |  |  |  |  |  |  |
| 22 | *L. longirostris* | 7.9–8.1 | 7.3–8.4 | 6.6 | 11.3 | 7.3 | 7.7 | 8.4 | 8.8 | 8.4 | 7.9 | 11.3 | 9.5 | 9.2 | 10.8 | 9.9 | 7.8 | 9.8 | 10.9 | 10.7 | 9.5 | 5.7–5.9 | – |  |  |  |  |  |  |  |
| 23 | *L. poecilochilus* | 9.4–9.8 | 8.7–10.2 | 7.9 | 12.9 | 10.2 | 9.6 | 10.5 | 9.8 | 9.6 | 9.8 | 12.2 | 11.3 | 9.4 | 11.1 | 9.8 | 8.3 | 11.4 | 10.1 | 12.1 | 10.7 | 6.8–8.3 | 8 | – |  |  |  |  |  |  |
| 24 | *L. fragilis* | 8–8.5 | 5.9–7.2 | 8.1 | 11.6 | 7.8 | 7 | 7.2 | 7.4 | 7.4 | 7.9 | 11.8 | 9.8 | 10.4 | 11.8 | 10.7 | 8.1 | 10.7 | 9 | 10.3 | 11.1 | 5.9–7.4 | 6.6 | 8.5 | – |  |  |  |  |  |
| 25 | *L. albilabris* | 7.2–7.6 | 9.5–10.2 | 7 | 10.7 | 7.7 | 7.7 | 8.3 | 8.3 | 7.9 | 7.4 | 10.5 | 9.2 | 10 | 11.2 | 9.6 | 7.5 | 9.3 | 9.3 | 11.1 | 9.6 | 5.7–6.8 | 6.8 | 6.5 | 7 | – |  |  |  |  |
| 26 | *L. laticeps* | 9.8–10 | 10.1–107 | 9.2 | 12.6 | 10.2 | 10 | 10.7 | 10.7 | 10 | 9.6 | 14.4 | 12.4 | 11.3 | 13.3 | 10.7 | 10.1 | 12.9 | 12.3 | 13 | 11.6 | 8.1–8.7 | 8.8 | 9.4 | 8.9 | 8.1 | – |  |  |  |
| 27 | *L. syphax* | 9.9–10.5 | 10.1–10.7 | 10.1 | 12 | 12.1 | 10.7 | 11 | 11.4 | 11.4 | 10.3 | 14.3 | 12.7 | 11.8 | 13.2 | 11.6 | 11.9 | 12.7 | 12.1 | 13.3 | 11.9 | 9.4–10.3 | 11.3 | 10.9 | 11.6 | 9.7 | 7 | – |  |  |
| 28 | *L. labosus* | 10–10.9 | 9.3–10.2 | 9.9 | 11.9 | 10.5 | 10.1 | 10.5 | 10.3 | 10.5 | 10.9 | 14.7 | 12.3 | 12 | 13.6 | 12.7 | 10.1 | 12.9 | 13 | 13 | 12.1 | 8.1–9 | 10.8 | 10.3 | 9.6 | 9.2 | 8.9 | 11.6 | – |  |
| 29 | *L. ventrimaculatus* | 10.2–11.1 | 9.6–10.4 | 10.1 | 12.6 | 10.3 | 10.8 | 11.2 | 11 | 11.2 | 11.6 | 15.4 | 12.9 | 11.8 | 13.4 | 12 | 10.5 | 13.1 | 13.2 | 13.7 | 12.5 | 8.5–9 | 10.6 | 10.9 | 10.3 | 9.4 | 8.5 | 11.6 | 0.6 | – |
