# Supplementary material for: A new frog of the Leptodactylus fuscus species group (Anura: Leptodactylidae), endemic from the South American Gran Chaco
Source: PeerJ. 2019 Oct 11;7:e7869. doi: 10.7717/peerj.7869 (PMC6791353; doi:10.7717/peerj.7869)
Supplement: Supplemental Information 8 [file peerj-07-7869-s008.rtf]

Leptodactylus mystacinus (non Burmeister, 1861): Acosta (2014); Álvarez et al. (2009); Aquino et al. (1996, partim); Baker & Vaucher (1984, partim); Berg (1986, partim); Bogart (1974, partim); Brusquetti & Lavilla (2006, partim); Bucher (1980); Cabral et al. (2017); Cei (1968; 1980, partim); Cruz et al. (1992); de la Riva (1990); de la Riva et al. (2000); de Sá et al. (2014, partim); Duellman (1999, partim); Eisentraut (1932); Fabrezi (2011, partim); Fernández et al. (2009, partim); Gallardo (1964, partim; 1966, 1979); Gallardo & Varela de Olmedo (1992, partim); Gans (1960); Gonzales (1998); Gonzales & Reichle (2002); Gonzales et al. (2006); Guerra & Aráoz (2016); Heyer (1969a, b, 1974, 1978 partim); Heyer & Diment (1974, partim); Heyer & Maxson (1982, partim); Heyer et al. (2003, partim); Jansen et al. (2011, 2016); Kacoliris et al. (2006); Köhler (2000); Köhler et al. (2017); Laurent & Terán (1982); Lavilla (2001); Lavilla et al. (1995; 1996; 2000a, partim; 2000b); Lobo (1995); Medina et al. (2013, 2016, partim); Méhely (1904, partim); Montero (1986); Motte et al. (2009, partim); Núñez & Weiler (2015); Peracca (1897); Pérez-Iglesias et al. (2017); Perotti (1994); Pinto-Viveros et al. (2017); Ponssa (2008, partim); Ponssa & Medina (2016, partim); Scott & Lovett (1975); Vaira et al. (2009; 2012, partim); Vellard (1948); Vera et al. (2018); Weiler et al. (2013, partim).

Leptodactylus cf. mystacinus: Lavilla & Manzano (1995); Perotti (1997).

References

Burmeister H. 1861. Reise durch die La Plata-Staaten mit besonderer Rücksicht auf die Physische Beschaffenheit und den Culturzustand der Argentinische Republik. Ausgefuhrt in den Jahren 1857, 1858, 1859 un 1860. Volume 2. Halle: HW Schmidt.
Acosta NR. 2014. Estado de conservación de los anfibios en la provincia de Salta: dimensión local de un problema global. Lhawet 3:35–40.
Álvarez BB, García JAR, Céspedez JA, Hernando AB, Zaracho VH, Calamante CC, Aguirre RH. 2009. Herpetofauna, provinces of Chaco and Formosa, Chaco Oriental region, north-eastern Argentina. Check List 5:74–82. 
Aquino AL, Scott NJ, Motte M. 1996. Lista de anfibios y reptiles del Museo Nacional de Historia Natural del Paraguay (Marzo, 1980-Septiembre, 1995). In Martínez OR, ed. Colecciones de Flora y Fauna del Museo Nacional de Historia Natural del Paraguay. San Lorenzo: Ministerio de Agricultura y Ganaderia, 331–400.
Baker MR, Vaucher C. 1984. Parasitic helminths from Paraguay VI: Cosmocerca Diesing, 1861 (Nematoda: Cosmocercoidea) from frogs. Revue Suisse de Zoologie 91:925–934.
Berg C. 1986. Batracios argentinos. Enumeración sistemática, sinonímica y bibliográfica de los batracios de la República Argentina (con un cuadro sinóptico de clasificación). Anales del Museo Nacional de Buenos Aires 5:147–226.
Bogart JP. 1974. A karyosystematic study of frogs in the genus Leptodactylus (Anura: Leptodactylidae). Copeia 3:728–737 DOI 10.2307/1442686.
Brusquetti F, Lavilla EO. 2006. Lista comentada de los anfibios de Paraguay. Cuadernos de Herpetología 20:3–79.
Bucher EH. 1980. Ecología de la fauna chaqueña. Una revisión. Ecosur 7:111–159.
Cabral H, Bueno-Villafañe D, Romero-Nardelli L. 2017. Comments on the diet of juvenile Erythrolamprus poecilogyrus caesius (Serpentes: Dipsadidae) in the Paraguayan Chaco. Phyllomedusa 16:299–302 DOI 10.11606/issn.2316-9079.v16i2p299-302.
Cei JM. 1968. Distribution et spécialisation des batraciens sudaméricains. In Delamare Deboutteville C, Rapoport E, eds. Biologie de l'Amérique Australe. Volume IV. Documents biogéographiques et écologiques, 199–214.
Cei JM. 1980. Amphibians of Argentina. Vol. 2. Firenze: Università degli Studi di Firenze.
Cochran DM. 1961. Type specimens of reptiles and amphibians in the U.S National Museum. Washington: Government Printing Office Washington.
Cruz FB, Perotti MG, Fitzgerald LA. 1992. Lista de anfibios y reptiles colectados en una localidad del Chaco Salteño. Acta Zoologica Lilloana 42:101–107.
de la Riva I. 1990. Lista preliminar comentada de los anfibios de Bolivia con datos sobre su distribución. Bollettino del Museo regionale di Scienze naturali, Torino 8:261–319.
de la Riva I, Köhler J, Lötters S, Reichle S. 2000. Ten years of research on Bolivian amphibians: updated checklist, distribution, taxonomic problems, literature and iconography. Revista Española de Herpetología 14:19–164.
de Sá RO, Grant T, Camargo A, Heyer WR, Ponssa ML, Stanley E. 2014. Systematics of the neotropical genus Leptodactylus Fitzinger, 1826 (Anura: Leptodactylidae): phylogeny, the relevance of non-molecular evidence, and species accounts. South American Journal of Herpetology 9(s1): S1–S100 DOI 10.2994/SAJH-D-13-00022.1 
Duellman WE. 1999. Distribution patterns of amphibians in South America. In: Duellman WE, ed. Patterns of Distribution of Amphibians. A Global Perspective. Baltimore: Johns Hopkins University Press, 255–328.
Eisentraut M. 1932. Biologische Beobachtungen im Bolivianischen Chaco. V. Ein Neuer Fall von Brutfürsorge in der Froschgattung Leptodactylus. Zeitschrift für Morphologie und Ökologie der Tiere 26:317–326.
Fabrezi M. 2011. Heterochrony in growth and development in anurans from the Chaco of South America. Evolutionary Biology 38:390–411.
Fernández M, Cole D, Heyer WR, Reichle, de Sá RO. 2009. Predicting Leptodactylus (Amphibia, Anura, Leptodactylidae) distributions: broad-ranging versus patchily distributed species using a presence-only environmental niche modeling technique. South American Journal of Herpetology 4:103–116 DOI 10.2994/057.004.0202.
Gallardo JM. 1964. "Leptodactylus prognathus" Boul. y "L. mystacinus"(Burm.) con sus respectivas especies Aliadas:(" Amphibia, Leptodactylidae" del grupo" Cavicola"). Revista del Museo Argentino de Ciencias Naturales 9:91–123.
Gallardo JM. 1966. Zoogeografía de los anfibios chaqueños. Physis 26:67–81.
Gallardo JM. 1979. Composición, distribución y origen de la herpetofauna chaqueña. The South American herpetofauna: its origin, evolution, and dispersal. Monographs of the Museum of Natural History of Kansas 7:1–485.
Gallardo JM, Varela de Olmedo E. 1992. Anfibios de la República Argentina: Ecología y comportamiento. Fauna de Agua Dulce de la República Argentina. Profadu (CONICET) 41:1–116.
Gans C. 1960. Notes on a herpetological collecting trip through the southeastern lowlands of Bolivia. Annals of Carnegie Museum 35:283–314
Gonzales L. 1998. La herpetofauna del Izozog. Ecología en Bolivia 31:45–52.
Gonzales L, Muñoz A, Cortez E. 2006. Primer reporte sobre la herpetofauna de la reserva natural “El Corbalán”, Tarija, Bolivia. Kempffiana 2:72–94.
Gonzales L, Reichle S. 2002. Singularidades de la herpetofauna de la serranía de Santiago de Chiquitos, Santa Cruz - Bolivia. Revista Boliviana de Ecología y Conservación Ambiental 11:77–85.
Guerra C, Aráoz E. 2016. Amphibian malformations and body condition across an agricultural landscape of northwest Argentina. Diseases of aquatic organisms 121:105–116 DOI 10.3354/dao03048.
Kacoliris FP, Berkunsky I, Williams J. 2006. Herpetofauna of the Argentinean Impenetrable Gran Chaco. Phyllomedusa 5:149–157.
Heyer WR. 1969a. Studies on the Genus Leptodactylus (Amphibia, Leptodactylidae): A Redefinition of the Genus Leptodactylus and a Description of a New Genus of Leptodactylid Frogs. Contributions in Science 155:1–14.
Heyer WR. 1969b. The adaptive ecology of the species groups of the genus Leptodactylus (Amphibia, Leptodactylidae). Evolution 23:421–428. 
Heyer WR. 1978. Systematics of the fuscus group of the frog genus Leptodactylus (Amphibia, Leptodactylidae). Natural History Museum of Los Angeles County 29:1–85.
Heyer WR, Diment MJ. 1974. The karyotype of Vanzolinius discodactylus and comments on usefulness of karyotypes in determining relationships in the Leptodactytus complex (Amphibia, Leptodactylidae). Proceedings of the Biological Society of Washington 87:327–336.
Heyer WR, Maxson LR. 1982. Distributions, relationships, and zoogeography of lowland frogs: The Leptodactylus complex in South America, with special reference to Amazonia. In: Prance GT, ed. Biological Diversification in the Tropics. New York: Columbia University Press, 375–388.
Heyer MM, Heyer WR, de Sá RO. 2003. Leptodactylus mystacinus. Catalogue of American Amphibians and Reptiles 767:1–11.
Jansen M, Bloch R, Schulze A, Pfenninger M. 2011. Integrative inventory of Bolivia's lowland anurans reveals hidden diversity. Zoologica Scripta 40:567–583 DOI 10.1111/j.1463-6409.2011.00498.x.
Jansen M, Masurowa A, O'Hara RB. 2016. Temporal variation, duty cycle, and absolute calling effort during sustained calling of Leptodactylus mystacinus (Anura: Leptodactylidae). Salamandra 52:328–336.
Köhler J. 2000. Amphibian diversity in Bolivia: a study with special reference to montane forest regions. Bonner zoologische Monographien 48:1–243.
Köhler J, Jansen M, Rodríguez A, Kok PJR, Toledo LF, Emmrich M, Glaw F, Haddad CFB, Rödel MO, Vences M. 2017. The use of bioacoustics in anuran taxonomy: Theory, terminology, methods and recommendations for best practice. Zootaxa 4251:1–124 DOI 10.11646/zootaxa.4251.1.1.
Laurent RF, Terán EM. 1982. Lista de los anfibios y reptiles de la Provincia de Tucumán. Miscelánea 71:1–15 
Lavilla EO. 2001. Amenazas, declinaciones poblacionales y extinciones en anfibios argentinos. Cuadernos de Herpetología 15:59–82.
Lavilla EO, Cruz FB, Scrocchi GJ. 1995. Amphibiens et reptiles de la station biologique Los Colorados dans la province de Salta, Argentine. II. Revue française d'aquariologie 22:117–128.
Lavilla EO, Gonzales L, Fernández I. 1996. Informe sobre la herpetofauna del Parque Nacional Amboró y areas aledañas. In: Rumiz D, ed. Componente Fauna del Plan de Manejo del Parque Nacional Amboró. Informe FAN-TNC. Santa Cruz de la Sierra. 
Lavilla EO, Manzano AS. 1995. La batracofauna de las selvas de montaña del Noroeste argentino. Reunión Regional Sobre Selvas de Montañas. Horco Molle, Tucumán (Argentina) 15–17.
Lavilla EO, Ponssa ML, Baldo D, Basso N, Bosso A, Céspedez, Chebez JC, 
Faivovich J, Ferrari L, Lajmanovich R, Langone JA, Peltzer P, Ubeda C, Vaira M, Vera Candioti F. 2000a. Categorización de los anfibios de Argentina. In: Lavilla EO, Richard E, Scrocchi JS, eds. Categorización de Los Anfibios y Reptiles de la República Argentina. San Miguel de Tucumán: Asociación Herpetologica Argentina, 11–34.
Lavilla EO, Vaira M, Ponssa ML, Ferrari L. 2000b. Batracofauna de las Yungas Andinas de Argentina: una síntesis. Cuadernos de Herpetología 14:5–26. 
Lobo F. 1995. Análisis filogenético del género Pseudopaludicola (Anura: Leptodactylidae). Cuadernos de Herpetología 9:21–33.
Medina RG, Ponssa ML, Aráoz E. 2016. Environmental, land cover and land use constraints on the distributional patterns of anurans: Leptodacylus species (Anura, Leptodactylidae) from Dry Chaco. PeerJ 4:e2605 DOI 10.7717/peerj.2605. 
Medina RG, Ponssa ML, Guerra C, Aráoz E. 2013. Amphibian abnormalities: Historical records of a museum collection in Tucuman Province, Argentina. The Herpetological Journal 23:193–202.
Méhely L. 1904. Investigations on Paraguayan batrachians. Annales Historico-Naturales Musei Nationalis Hungarici 2:207–232.
Montero R. 1986. Porcentaje de catecolaminas adrenales como indicador filogenético en Leptodactylidae e Hylidae. Cuadernos de Herpetología 2:1–10.
Motte M, Núñez K, Cacciali P, Brusquetti F, Scott N, Aquino AL. 2009. Categorización del estado de conservación de los anfibios y reptiles de Paraguay. Cuadernos de Herpetología 23:5–18.
Núñez K, Weiler A. 2015. Ensamble de anuros de la estancia montanía en el Chaco Seco (Boquerón, Paraguay). Reportes Científicos de la FACEN 6:41–44.
Peracca MG. 1897. Viaggio del Dott. Alfredo Borelli nel Chaco boliviano è nella Repubblica Argentina. Rettili ed Anfibi. Bollettino dei Musei di Zoología ed Anatomía Comparata délia R. Università di Torino 12:1–19.
Pérez-Iglesias JM, Jofré LE, Rueda MP. 2017. Primeros registros de la herpetofauna en dos áreas naturales protegidas de la provincia de Santiago del Estero (Argentina). Cuadernos de Herpetología 31:49–57.
Perotti MG. 1994. Aportes preliminares sobre la reproducción en una comunidad de anuros chaqueños en Argentina. Cuadernos de Herpetología 8:39–50.
Perotti MG. 1997. Modos reproductivos y variables reproductivas cuantitativas de un ensamble de anuros del Chaco semiárido, Salta, Argentina. Revista Chilena de Historia Natural 70:277–288.
Pinto-Viveros MA, Mano-Cuellar K, Escalante RS, Villarroel D, Pinto-Ledezma JN. 2017. Historia natural del Cerro Mutún: IV. La herpetofauna. Kempffiana 13:106–118.
Ponssa ML. 2008. Cladistic analysis and osteological descriptions of the frog species in the Leptodactylus fuscus species group (Anura, Leptodactylidae). Journal of Zoological Systematics and Evolutionary Research 46:249–266 DOI 10.1111/j.1439-0469.2008.00460.x.
Ponssa ML, Medina RG. 2016. Comparative morphometrics in Leptodactyline Frogs (Anura, Leptodactylidae, Leptodactylinae): does burrowing behavior relate to sexual dimorphism? Journal of Herpetology 50:604–615 DOI 10.1670/15-156.
Scott NJ, Lovett. JW. 1975. A collection of reptiles and amphibians firom the Chaco of Paraguay. The University of Connecticut Occasional Papers, Biological Science Series 2:257–266.
Vaira M, Akmentins M, Attademo M, Baldo D, Barrasso DA, Barrionuevo S, Basso N, Blotto B, Cairo S, Cajade R, Céspedez J, Corbalán V, Chilote P, Duré M, Falcione C, Ferraro D, Gutierrez FR, Ingaramo MR, Junges C, Lajmanovich R, Lescano JN, Marangoni F, Martinazzo L, Marti R, Moreno L, Natale GS, Pérez Iglesias JM, Peltzer P, Quiroga L, Rosset S, Sanabria E, Sanchez L, Schaefer E, Úbeda C, Zaracho V. 2012. Categorización del estado de conservación de los anfibios de la República Argentina. Cuadernos de Herpetología 26:131–159.
Vaira M, Muñoz A, Gonzáles L. 2009. Anfibios de la Selva Pedemontana de Argentina y Bolivia. In: Brown AD, Blendinger PG, Lomáscolo T, García Bes P, eds. Selva pedemontana de las Yungas: historia natural, ecología y manejo de un ecosistema en peligro. Buenos Aires: Ediciones del Subtrópico, 214–233.
Vellard JEAN. 1948. Batracios del chaco argentino. Acta Zoologica Lilloana 5:137–174.
Vera MC, Abdala V, Aráoz E, Ponssa ML. 2018. Movement and joints: effects of overuse on anuran knee tissues. PeerJ 6:e5546.
Weiler A, Nuñez K, Airaldi K, Lavilla E, Peris S, Baldo D. 2013. Anfibios del Paraguay. Asunción: Facultad de Ciencias Exactas y Naturales.
